# Trends in social determinants of inequality in child undernutrition from the Ethiopian Demographic and Health Surveys, 2005–2016

**DOI:** 10.1371/journal.pone.0295810

**Published:** 2024-01-12

**Authors:** Frehiwot Birhanu, Kiddus Yitbarek, Evan Atlantis, Mirkuzie Woldie, Firew Bobo

**Affiliations:** 1 School of Public Health, College of Health Sciences, Mizan-Tepi University, Mizan Aman, Southwest Ethiopia; 2 Department of Health Policy and Management, Institute of Health, Jimma University, Jimma, Ethiopia; 3 School of Health Sciences, Western Sydney University, Penrith, New South Wales, Australia; 4 Fenot Project, School of Population and Public Health, University of British Columbia, Addis Ababa, Ethiopia; 5 School of Public Health, University of Technology Sydney, Sydney, New South Wales, Australia; University/College Library, ETHIOPIA

## Abstract

**Background:**

While child undernutrition has been eliminated in some middle-income countries, it remains highly prevalent in sub-Sahara African (SSA) and South Asian regions, and is disproportionately concentrated among the poor. In this study, we estimated trends in child undernutrition by social determinants and related risks from wealth inequality in Ethiopia, from 2005 to 2016.

**Method:**

We analyzed data from three consecutive surveys (2005, 2011, and 2016) from the Ethiopian Demographic and Health Survey. First, we estimated trends in the prevalence of childhood undernutrition variables (stunting, underweight, and wasting) and social determinants (household wealth status, education level, place of residence, and administrative regions). Then we assessed evidence of undernutrition by wealth-related inequality with concentration curves (visual) and concentration indeces (quantitative). A multilevel mixed-effect Poisson regression model was used to identify predictors of undernutrition variables expressed as covariate-adjusted rate ratios, with 95% confidence intervals (RRs, 95%CI).

**Result:**

A total of 23,934 mother-child pairs were obtained from the three surveys. The average prevalence decreased by 12.4 percentage points for stunting (from 50.8 to 38.4%, P<0.01), 9.5 percentage points for underweight (33.2% to23.7%, P<0.01), and 2.1 percentage points for wasting (12.2% to10.1%, P<0.01). There was persistent and statistically evidence of wealth inequality in stunting, underweight, and wasting (concentration indeces of -0.2 to -0.04, all P values <0.05). Stunting, underweight, and wasting variables were associated with male sex of the child (RR 0.94, 0.95, 0.85, all P-values <0.01) recent diarrhea (RR 1.18, 1.27, 1.37, all P-values <0.01), secondary education status of the mother (RR 0.66, 0.57, 0.61, all P-values < 0.057), increasing wealth index (richest) (RR 0.73, 0.70, 0.50, all P-values < 0.05), and having no toilet facility (RR 1.16, 1.22, 1.18, all P-values < 0.05).

**Conclusion:**

Despite the decreased burden of stunting and underweight, the prevalence of wasting remained relatively unchanged in Ethiopia from 2005 to 2016. Moreover, wealth-related inequality in child undernutrition increased for most of the child undernutrition indicators during this period. Social determinants of child undernutrition warrant urgent implementation of strategies to reduce their health impacts in SSA.

## Introduction

Child undernutrition is almost non-existent in the World Health Organization (WHO) regions of Europe and Central Asia; Latin America and the Caribbean countries [1]. However, the decline in sub-Saharan Africa (SSA) and South Asian countries is less encouraging and somewhat complex [2]. In developing countries, it is estimated that 178, 112, and 55 million children under five have stunting, underweight, and wasting, respectively [3, 4]. Consequently, child undernutrition is a leading cause of preventable child mortality in this region [5, 6].

Child undernutrition is associated with a range of health consequences across the short, medium, and long terms [7]. Infectious diseases like diarrhea and pneumonia increase the risk of death in the short term for undernourished infants and children [8, 9]. Undernutrition increases the risk of cognitive development in the medium term, resulting in intergenerational and entrenched educational disadvantages [10, 11]. Early life, in utero to the first year of postnatal life, undernourishment has long-term consequences for survivors, thereby increasing their risk of non-communicable diseases (NCD) later in life [12]. Pathological mechanisms hypothesized include the ‘capacity-load’ model, which posits the first 1,000 days of life to be a critical time for developing metabolic capacity [13, 14].

Evidence indicates that child undernutrition in all forms is concentrated among the poorest groups of the population [4, 15]. To minimize unnecessary and avoidable differences in health, the United Nations (UN) launched the Sustainable Development Goals (SDGs) in 2012 targeting the inclusion of the disadvantaged population with a principle of leaving no one behind. Goal 2, 3, and 10 of the seventeen goals argues for ending hunger, promoting well-being, and reducing inequality both within and among countries. Despite this call, studies suggest that the progress achieved in recent decades has stalled and inequality within and across countries has increased recently [16–18].

The economic status of the household where the children belong is a common factor that imposes differences in the child’s nutritional status within the country [19, 20]. Various studies reported children from economically better-off families to have a good status in their anthropometry [21–23]. Furthermore, characteristics like the educational status of the mother, place of residence (rural/urban), and the number of siblings is directly related to inequality of the child’s undernutrition [24].

In Ethiopia, a SSA country, the report from the Demographic and Health Survey (DHS) showed mortality among children under five decreased from 166 to 67 per 1000 live births between 2000 and 2016; infant mortality decreased from 97 to 48 per 1,000 live births between 2000 and 2016; whereas neonatal death remained relatively stable [25–28]. Moreover, understanding of trends in the level of inequality in child undernutrition is still unclear as most of the studies in Ethiopia focused on specific geographical areas, points in time, or examining determinants for child undernutrition [29–31]. To address this knowledge gap, this study examined to interrogate trends in child undernutrition and related predictors in Ethiopia between 2005 and 2016.

## Methods

The present study is reported according to the international guideline of Strengthening the Reporting of Observational Studies in Epidemiology (STROBE) checklist for reporting cross-sectional studies [32].

### Study design, and setting

We conducted this study using the three waves of Ethiopian DHS data (2005, 2011, and 2016). Ethiopia is Africa’s oldest independent country located in the Eastern part of Africa. Administratively it is composed of 11 regions, of which seven are predominantly agricultural, two pastoralists (livestock raising as the main way of living), two regions with both agrarian and pastoralist areas, and two chartered cities [33]. The population of Ethiopia is around 110 million with nearly 16% shared by children under the age of five [34]. Almost all of the population rely on rain-fed agriculture for survival [35]. The health system is decentralized into the eleven administrative regions and the two city-administration. Access to health care is an exigent challenge in Ethiopia, where there are only 353 functional hospitals, 3,735 health centres, and 17,550 health posts in 2020 [36, 37]. In addition, according to recent information the health professional (Medical Doctors, Midwives, and Nurses) to population ratio is 1.81 per one thousand population in the year 2019, which is below the minimum requirement for SSA [38].

### Data source

In this study, three consecutive survey data, 2005, 2011, and 2016, of`Ethiopian DHS were accessed and used from the MEASURE DHS program database (The DHS Program—Ethiopia: Standard DHS, 2005, The DHS Program—Ethiopia: Standard DHS, 2011, and The DHS Program—Ethiopia: Standard DHS, 2016) respectively. The survey team used DHS Program’s standard tools that were adapted to reflect the population and health issues relevant to Ethiopia. Among all the five modules in the DHS program, we used the children’s data collected with the women’s questionnaire. The questionnaire includes items related to respondents’ background characteristics, reproduction, contraception, pregnancy and postnatal care, child nutrition, childhood immunizations, and health facility information. The data were collected electronically using tablet computers. The Survey was implemented by the Ethiopian Public Health Institute (EPHI), in partnership with the DHS Program, the Central Statistical Agency (CSA), and the Ethiopian Ministry of Health (MOH).

### Participants

In all three consecutive surveys, the samples were taken in two stages. In the first stage, from the list of enumeration areas (EAs) created for the Ethiopian population and housing census, about 540 EAs were taken in 2005, 624 EAs in 2011, and 645 EAs in 2016. An EA is a geographic area covering an average of 131 households. Household listing was conducted in all the selected enumeration areas before sampling the households. In the second stage, a fixed number of sample households were taken from each of the selected enumeration areas. The source population in this study was mother-child pairs of 4306 in 2005, 10,040 in 2011, and 9588 in 2016 yielding a total of 23,934 mother-child pairs [Table 1].

**Table 1:** Sampling procedure for the Ethiopian Demographic and Health Surveys.

	2005	2011	2016
Sampling frame	58,702	85,057	84,915
Enumeration areas	540 (145 urban and 395 rural)	624 (187 in urban areas and 437 in rural)	645 EAs (202 in urban areas and 443 in rural areas)
Mother-to-child pairs	4,306	10,040	9588

### Variables and measurement

#### Measurements

The dependent variables were stunting, underweight, and wasting. The DHS methods measured weight using a solar-powered digital scale (Seca 878) measururing in 0.1 kg increments. Height measurements are carried out using a measuring board while lying down for children younger than 24 months and while standing for older children. Classifications of child undernutrition were based on WHO Child Growth Standards. The undernutrition indicators were measured as: ‘stunting’ if the height-for-age Z score is less than -2 standard deviations (SDs); ‘wasting’ if the weight for height Z score is less than -2SDs, and similarly ‘under-weight’ if the weight-for-age Z score is less than -2SDs [39]. We then rated the child as ‘undernourished’ if they had at least one of the abovementioned undernutrition indicators. Severe undernutrition was defined as having a Z score less than -3SDs for stunting, underweight, and wasting.

#### Independent variables

We used a framework developed by the WHO Commission on Social Determinants of Health to explain determinants of inequality in stunting, underweight, and wasting. Household wealth index and educational status were considered to determine the socioeconomic position of child-mother pairs. We used variables like sex of the child, child age in months, birth order, the occurrence of cough or breathing problem, diarrhea, age of the mother, mother’s educational status, household wealth status, a major source of drinking water, type of toilet facility, lack of money for treatment, distance to health facility and place of residence as independent predictors. The DHS program uses the wealth index to measure and show the household’s living standard [40]. The wealth index was calculated using data on the household’s ownership of selected assets, and materials then the final wealth quintile was obtained after a valuation and analysis using principal component analysis. The households accordingly fall in either of the poorest, poor, middle, rich, or richest quantile.

#### Bias

To minimize the possible bias, missing responsecategories like “don’t know”, “Missing”, inconsistent” were excluded when calculating basic statistics during data analysis. In addition, all analyses were adjusted for cluster and sampling weights for disproportionate stratification of recruited participants.

### Statistical analysis

We first estimated trends of stunting, underweight, and wasting from 2005 to 2016 using percentage points. We then calculated rate differences to examine changes from 2005 to 2016 in stunting, underweight, and wasting by education level, household wealth status, place of residence, and administrative regions. We used concentration indeces and concentration curves to assess the economic inequality in child nutritional status. Concentration curves were plotted considering the cumulative percentage of child undernutrition (y-axis) against the cumulative percentage of the population, ranked by socio-economic variables (x-axis). To quantify equity differences, concentration index values were computed with the respective 95% confidence interval. The concentration index is twice the area between the concentration curve and the line of equality (the 45-degree line). The concentration index value ranges from -1 to +1. The convention is when the index value is negative the curve lies above the line of equality, indicating the disproportionate concentration of child undernutrition among the poor, and when it is positive it lies below the line of equality, showing the disproportionate child undernutrition among the rich [41, 42].

The DHS program uses a multistage cluster sampling technique where participants in the survey are nested within Primary Sampling Units (PSU) [43]. We used a Multilevel mixed effect poisson regression model to adjust for the hierarchical nature of the data–at level one we adjusted mother-to-child pairs and at level two we adjusted for primary sampling units (clusters). We started the model-building process with the unconditional model (a model containing no predictors), and then more complex models were built gradually by checking improvements in model fit after each model was estimated. We used a Generalized Latent Linear Mixed Model (GLLMM) in Stata, which enabled us to adjust for the hierarchical nature of the data and the sampling weights.

We used pooled data from all three surveys of the Ethiopian DHS to examine predictors of inequality in stunting, underweight, and wasting. The pooled regression analysis is adjusted for the selected covariates. Results are presented with adjusted rate ratios (RRs) and statistical significance was declared when the P-value was <0.05. Analyses were conducted using Stata 14.2.

### Ethical considerations

The data for this study were obtained from the DHS program, For our purpose, we formally requested and completed the agreement and data usage form before proceeding with the analysis. There was no additional ethical approval sought by the authors.

## Results

With a response rate of 96%, 95%, and 95.1% for the years 2005, 2011, and 2016 respectively, this analysis includes information collected from 4,306 mother-child pairs in 2005, 10,040 in 2011, and 9,588 in 2016, yielding a total of 23,934 mother-child pairs. The majority (89%) of the women were from rural areas, and more than 42% were from Oromia, the largest regional state in the country. The highest proportion (27.8%) of the children were sixth or above by birth order and 51% of the children were male. The majority of (69.3%) the mothers had no education at all. More than one-fifth (22%) of the women included in the analysis were either from the poorest or poorer families. Moreover, the majority (68.7%) of women mentioned lack of money for treatment as a big problem (Table 2).

**Table 2:** The prevalence of undernourished children under five by household wealth status, educational status of mothers, place of residence, and regions in Ethiopia (DHS 2005 to 2016).

Study characteristics	Prevalence (%) of stunting			% change during 2005–2016	Prevalence (%) of underweight			% change during 2005–2016	Prevalence (%) of wasting			% change during 2005–2016
	2005	2011	2016		2005	2011	2016		2005	2011	2016	
**Mother’s education**												
No education	53.3	46.6	41.6	11.7	36.0	31.3	27.4	8.6	13.2	10.9	10.9	2.3
Primary	45.3	41.8	35.3	10	26.4	25.2	18.1	8.3	9.9	7.9	8.9	1.0
Secondary or higher	29.7	19.9	20.2	9.5	11.3	8.9	11.0	0.3	4.1	3.9	7.5	-3.4
**Wealth status**												
Poorest	52.7	48.8	45.1	7.6	36.2	35.8	30.9	5.3	14.4	12.6	14.0	0.4
Poorer	55.0	47.6	43.1	11.9	39.2	32.9	27.4	11.8	16.2	12.3	9.8	6.4
Middle	52.2	46.0	37.7	14.5	33.3	29.1	23.2	10.1	12.3	9.5	10.4	1.9
Richer	51.0	45.2	34.7	16.3	29.7	26.0	17.2	12.5	8.6	7.8	7.0	1.6
Richest	40.2	29.1	25.5	14.7	24.5	15.1	15.0	9.5	7.8	5.1	7.8	0.0
**Place of residence**												
Urban	35.6	31.3	26.1	9.5	17.4	16.7	14.1	3.3	8.6	5.6	9.3	-0.7
Rural	52.1	46.2	39.9	12.2	34.5	30.5	24.9	9.6	12.5	10.5	10.2	2.3
**Regions**												
Tigray	47.2	51.0	38.8	8.4	36.2	35.3	22.6	13.6	13.6	10.5	11.4	2.2
Affar	41.9	50.0	40.7	1.2	32.6	40.6	36.2	-3.6	16.3	20.6	18.3	-2.0
Amhara	63.7	51.9	47.2	16.5	43.0	33.8	29.1	13.9	17.6	10.2	10.0	7.6
Oromia	44.2	41.5	36.3	7.9	29.1	26.0	22.5	6.6	9.9	9.8	10.6	-0.7
Somali	50.0	32.5	27.0	23	43.7	33.1	28.0	15.7	23.6	21.2	23.1	0.5
Benishangul-Gumuz	44.2	48.2	42.6	1.6	41.9	32.2	34.6	7.3	20.9	9.6	10.9	10.0
SNNPR	54.6	43.7	39.1	15.5	29.7	28.1	21.7	8	8.8	7.7	6.2	2.6
Gambela	36.4	29.0	22.7	13.7	18.2	22.6	18.2	0	9.1	12.9	13.6	-4.5
Harari	44.4	27.3	30.0	14.4	22.2	21.7	20.0	2.2	11.1	9.1	10.0	1.1
Addis Ababa	23.3	22.3	14.7	8.6	10.0	6.7	5.2	4.8	3.3	5.0	3.8	-0.5
Dire Dawa	33.3	34.4	41.7	-8.4	26.7	28.1	27.0	-0.3	13.3	12.5	10.8	2.5
**Average**	**50.8**	**44.3**	**38.4**	**12.4**	**33.2**	**28.8**	**23.7**	**9.5**	**12.2**	**9.9**	**10.1**	**2.1**

SNNPR; Southern Nations, Nationalities, and Peoples Region

### Trends of childhood undernutrition

**Fig 1:**
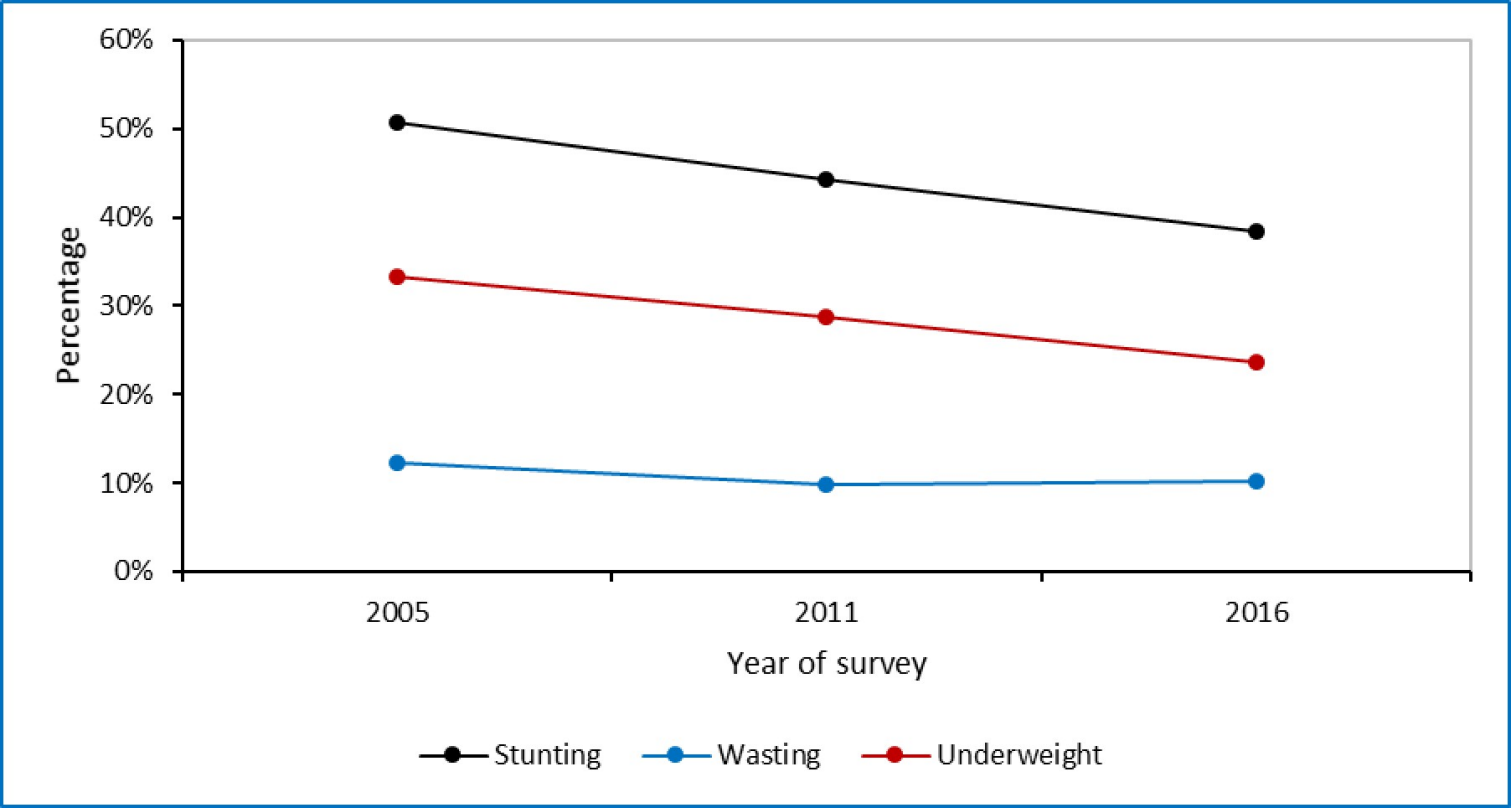
Trends in prevalence of stunting, underweight, and wasting among children under five in Ethiopia by survey year.

The prevalence of stunting decreased by 12 percentage points (from 50.8% in 2005 to 38.4% in 2016, P<0.01), while the prevalence of underweight decreased by 10 percentage points (from 33.2% in 2005 to 23.7% in 2016, P<0.01). The prevalence of wasting was found to be steady in the three consecutive surveys with a marginal decline by 2 percentage points (from 12.2%% in 2005 to 10.1% in 2016, P<0.01) [Fig 1].

The largest decline in child undernutrition rate was observed among children whose mothers had no education compared to mothers who had primary or higher education. For instance, for mothers who do not have an education; children stunting declined by 11.7, underweight by 8.6, and wasting by 2.3 percentage points, while the decline was 9.5, 0.3, and -3.4 percentage points for children of mothers having higher education. Trends of childhood undernutrition showed a larger decline among rural residents compared to urban residents. As compared to the poorest family, for women of the richest households the prevalence of stunting and underweight decreased by 14.7and 9.5 percentage points, respectivley from 2005 to 2016. Among regional states of Ethiopia, the Amhara regional state showed the largest decline in stunting prevalence by 16.5 percentage points, in the Somali region prevalence of underweight decreased by 1.7 percentage points, while Benishangul-Gumuz reduced wasting by 10.0 percentage points [Table 2].

### The socio-economic inequality in child undernutrition

**Fig 2:**
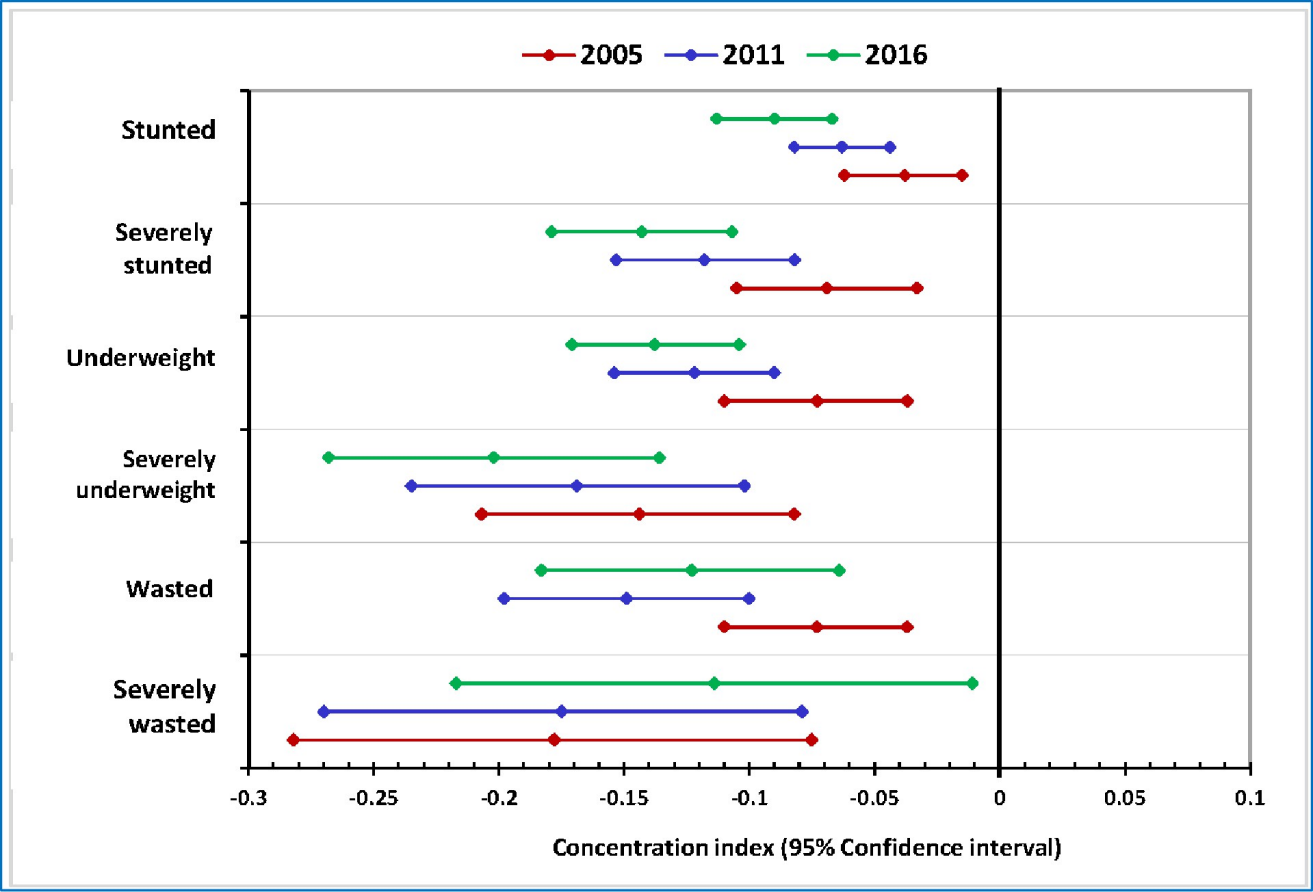
Concentration indices that show socioeconomic inequalities in child undernutrition in Ethiopia (DHS 2005 to 2016).

The negative values of concentration indices indicate the disproportionate concentration of stunting, underweight, and wasting among children from poor households in 2005, 2011, and 2016 (Fig 2). The inequality in stunting, underweight, and wasting was also statistically significant.

**Fig 3:**
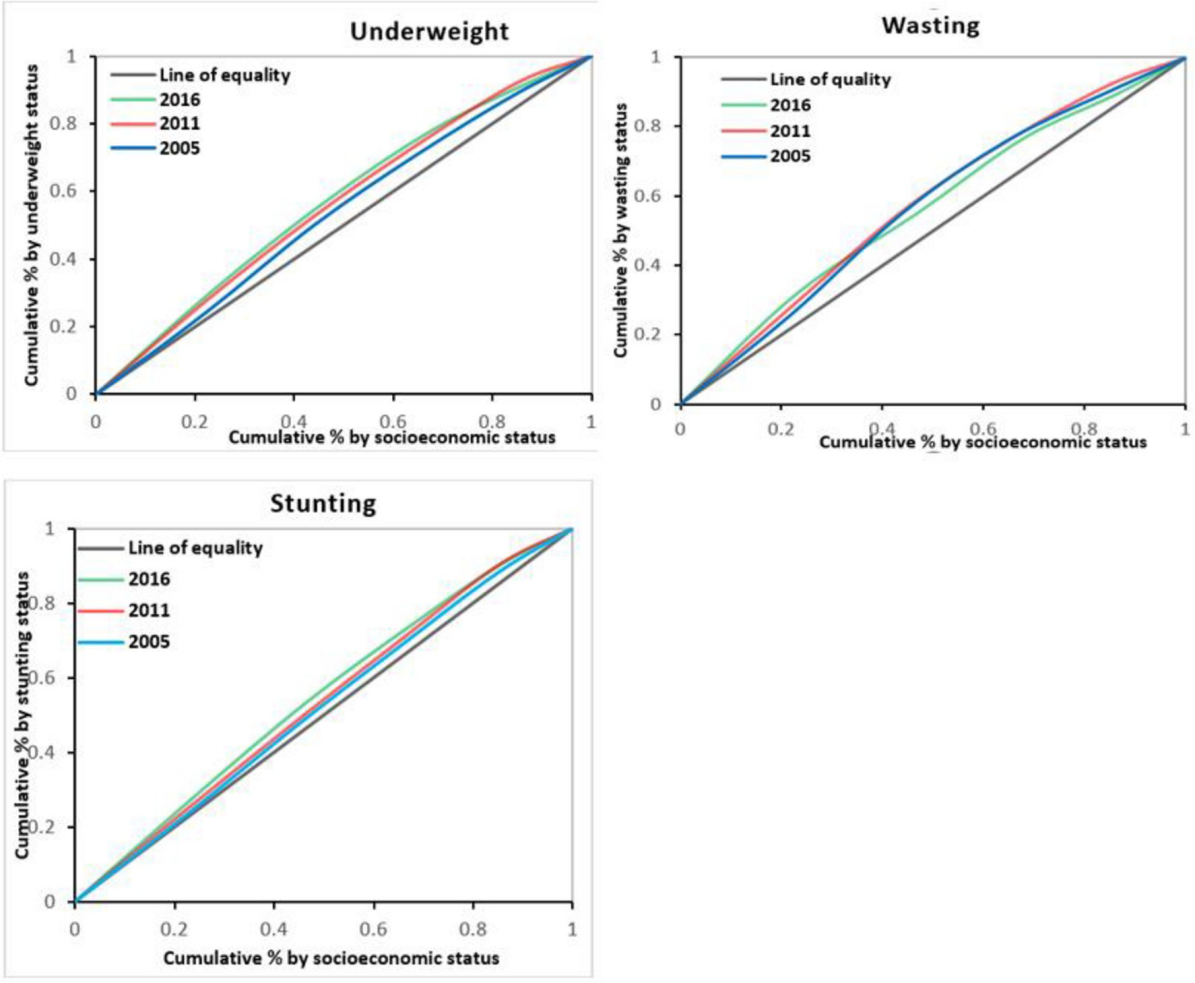
Concentration curve that shows socioeconomic inequalities in child undernutrition in Ethiopia (DHS 2005 to 2016).

The highest concentration index in stunting was observed in 2016 (-0.09) and the lowest was observed in 2005 (-0.01. The same trends were observed for severe stunting, underweight, and severe under-weight. Inequality increased across the eleven years between 2005 and 2016. Concerning wasting and severe wasting, on the contrary, there was a decreasing trend of inequality between 2005 and 2016. Overall, the concentration curves and the distribution of the concentration indices indicate that the inequality gap in child undernutrition was widening over the ten years covered by the surveys [Figs 2 and 3].

### Predictors of inequalities in undernutrition

The multilevel Poisson regression revealed several predictors of child undernutrition. Considering other variables constant, the wealth of the household hasd a significant inverse effect on stunting, underweight, and wasting with a strong association in underweight and wasting. Similarly, the mother’s level of education was found to have a significant inverse effect on the child’s undernutrition in all three measures. Place of residence urban versus rural, on the other hand, was negatively associated with stunting and wasting that approached statistical significance. Male sex of the child was associated with stunting, underweight, and wasting measures of undernutrition [Table 3].

**Table 3:** Multilevel mixed-effect Poisson regression: Pooled data (DHS: 2005 to 2016).

Variables	Category	Stunting		Underweight		Wasting	
		IRR (95% CI)	SE	IRR (95% CI)	SE	IRR (95% CI)	SE
Sex of the child	Male	**Reference**		**Reference**		**Reference**	
	Female	0.94 (0.9,0.99)*	0.02	0.95 (0.91,1)*	0.02	0.85 (0.78,0.93)**	0.04
Child age in months	< 12 months	**Reference**		**Reference**		**Reference**	
	12–23 months	4.17 (3.74,4.66)**	0.24	3.29 (2.98,3.65)**	0.17	1.89 (1.66,2.15)**	0.12
	23–35 months	4.18 (3.74,4.66)**	0.24	3.5 (3.17,3.87)**	0.18	1.11 (0.97,1.29)	0.08
	35–47 months	4.48 (4.02,5)**	0.25	3.0 (2.71,3.32)**	0.16	0.83 (0.71,0.97)*	0.07
	47–59 months	4.24 (3.79,4.74)**	0.24	2.99 (2.7,3.32)**	0.16	0.97 (0.83,1.13)	0.08
Birth order	First child	**Reference**		**Reference**		**Reference**	
	Second child	1.04 (0.95,1.13)	0.05	1.03 (0.94,1.13)	0.05	1.0 (0.85,1.18)	0.08
	Third child	1.09 (0.99,1.2)	0.05	1.19 (1.08,1.32)**	0.06	1.2 (1.01,1.43)*	0.11
	Fourth child	1.13 (1.02,1.25)*	0.06	1.26 (1.14,1.41)**	0.07	1.33 (1.1,1.61)**	0.13
	Fifth child	1.12 (1,1.26)*	0.06	1.26 (1.12,1.42)**	0.07	1.43 (1.16,1.75)**	0.15
	Sixth or above	1.17 (1.05,1.31)**	0.07	1.31 (1.17,1.47)**	0.08	1.41 (1.15,1.72)**	0.14
Child had cough recently	No	**Reference**		**Reference**		**Reference**	
	Yes	1.02 (0.96,1.09)	0.03	1.06 (1,1.14)	0.03	1.1 (0.98,1.23)	0.06
Child had diarrhea recently	No	**Reference**		**Reference**		**Reference**	
	Yes	1.18 (1.1,1.26)**	0.04	1.27 (1.19,1.36)**	0.04	1.37 (1.22,1.53)**	0.08
Maternal age	15–24	**Reference**		**Reference**		**Reference**	
	25–29	0.96 (0.88,1.03)	0.04	0.86 (0.79,0.93)**	0.03	0.82 (0.71,0.94)*	0.06
	30–34	0.92 (0.83,1.01)	0.04	0.83 (0.75,0.91)**	0.04	0.73 (0.61,0.87)**	0.06
	35–49	0.88 (0.8,0.98)*	0.05	0.78 (0.7,0.87)**	0.04	0.71 (0.59,0.86)**	0.07
Mother’s educational status	No education	**Reference**		**Reference**		**Reference**	
	Primary	0.91 (0.86,0.97)*	0.03	0.87 (0.81,0.93)**	0.03	0.85 (0.75,0.95)*	0.05
	Secondary and above	0.66 (0.57,0.77)**	0.05	0.57 (0.49,0.67)**	0.05	0.61 (0.46,0.79)**	0.08
Household wealth index	Poorest	**Reference**		**Reference**		**Reference**	
	Poorer	1.08 (1.01,1.15)*	0.04	0.99 (0.93,1.06)	0.04	0.81 (0.71,0.92)**	0.05
	Middle	0.96 (0.88,1.03)	0.04	0.91 (0.84,0.99)*	0.04	0.83 (0.72,0.96)*	0.06
	Richer	0.90 (0.82,0.98)*	0.04	0.78 (0.71,0.86)**	0.04	0.68 (0.58,0.81)**	0.06
	Richest	0.73 (0.65,0.83)**	0.05	0.70 (0.61,0.79)**	0.05	0.50 (0.39,0.63)**	0.06
Major source of drinking water	Unimproved	**Reference**		**Reference**		**Reference**	
	Improved	1.01 (0.95,1.06)	0.03	1.05 (1,1.11)	0.03	1.06 (0.96,1.17)	0.05
Type of toilet facility	Improved	**Reference**		**Reference**		**Reference**	
	Unimproved	1.25 (1.14,1.38)**	0.06	1.2 (1.09,1.33)**	0.06	0.9 (0.75,1.07)	0.08
	No facility	1.16 (1.05,1.29)**	0.06	1.22 (1.1,1.35)**	0.06	1.18 (0.99,1.41)	0.11
Lack of money for treatment	Is big problem	**Reference**		**Reference**		**Reference**	
	Not a big problem	0.99 (0.94,1.05)	0.03	0.97 (0.91,1.03)	0.03	0.94 (0.85,1.05)	0.05
Distance to health facility	Is big problem	**Reference**		**Reference**		**Reference**	
	Not a big problem	0.99 (0.93,1.05)	0.03	0.96 (0.91,1.03)	0.03	0.97 (0.86,1.08)	0.06
Place of residence	Urban	**Reference**		**Reference**		**Reference**	
	Rural	1.21 (1.07,1.36)**	0.07	1.12 (1,1.27)	0.07	0.77 (0.63,0.95)*	0.08
Survey year	2005	**Reference**		**Reference**		**Reference**	
	2011	0.93 (0.86,1)	0.04	1.05 (0.97,1.14)	0.04	1 (0.87,1.16)	0.07
	2016	0.78 (0.72,0.85)**	0.03	0.92 (0.84,1)	0.04	1.07 (0.92,1.24)	0.08
	_cons	0.08 (0.06,0.1)	0.01	0.1 (0.08,0.12)	0.01	0.13 (0.09,0.18)	0.02

*p-value < 0.05

** p-value < 0.01

## Discussion

This study confirms that child undernutrition rates have decreased between 2005 and 2016 in Ethiopia. While childhood stunting and underweight rates declined considerably during this period, there was a modest decline in the prevalence of wasting. This finding is similar to a systematic review in Ethiopia which found the prevalence of wasting declined slightly between 1997 and 2015 [44]. The possible reason for the persistent wasting might be associated with the Ethiopian recurrent history of conflict-led hunger and famine imposed by lack of access to appropriate food and unconducive enviromet to work [45, 46].

Despite this, child undernutrition appears to be high in other SSA countries as well, but is worst in East and Western Africa [47–49]. Although this region is known to be very fertile for agriculture and has the potential to produce enough food for local consumption and export to the rest of the world [50], it expriences ongoing food shortages attributable to the effects of climate change and repeated droughts [51]. The cyclic loop of undernutrition that starts in utero will continue to adulthood and give rise to another undernourished child, which impedes cognitive and educational performance at school and resulting lower economic productivity in the long run; this facilitates social and economic challenges in the country [52].

Though the progress in the reduction of child undernutrition was encouraging, the observed reduction was in favor of the wealthier households in all three surveys. The level of stunting, underweight, and wasting were concentrated among the poor subgroups of the population over a decade. A study conducted in Zimbabwe also revealed that the nutritional status inequality is widening over time [53]. In some other African countries, there is a positive change in equity of child nutrition favoring the poor population. A study in sub-Saharan African countries indicates countries like Cote d’Ivoire, Benin, Senegal, and Kenya considerably reduced the equality gap in childhood stunting over time [23].

Our analysis revealed that inequality in child undernutrition related to household wealth status has been increasing over time in many of the indicators except for severe wasting. For instance, the concentration index of stunting has reduced from -0.04 in 2005 to -0.09 in 2016 indicating a 44% increase in the concentration of stunting among the poor. The same applies to the underweight, in which the concentration of underweight among the poor was raised by about 50% over ten years. The concentration of wasting among the poor on the other hand had a fluctuating trend. It has been increasing between 2005 and 2011 and again dropped after 2011. This finding is in line with a study conducted in Nigeria in 2020, and East and Southern Africa region in 2022 indicating worsening of pro-rich inequalities in undernutrition indicators over time [54, 55].

Controlling other variables, our Poisson regression model also confirmed that wealth is among the main determinants of undernutrition in Ethiopia across all three measures of undernutrition. This might be due to the economic capacity of the households to ensure their food security and to access or afford health services whenever the child is ill [56, 57]. Furthermore, the other relevant socio-economic variable identified was the sex of the baby. In our study, male children were more prone to undernutrition, this finding was consistent with other studies conducted in Ethiopia, and other SSA countries [58–60]. The result might be attributable to the sex-based biological differenes in immune system development, which makes boys more vulnerable to recurrent infection that leds to undernourishment at early ages [61]. There is also evidence suggesting that boys are more likely to be born as a preterm than female [62, 63] which increase adverse event in the early life leaving a negative sequel regarding male child nutritional and health outcomes.

Place of residence was the other predictor variable, in this study children living in the rural area had higher stunting and wasting as compared to their urban counterparts. This study is supported by studies conducted in Ethiopia, and other LMICs [63, 64]. This possible reason for this might be the relatively favourable parental charactersthics at urban area. Most of the time parents at urban area are more likely to be educated, and have a better economical stand to afford food items as compared to the rural households [65].

We also found that maternal educational status was a critical driver of undernutrition across all three measures. Other several studies found the same finding indicating wealth, education, and place of residence to be a determinant of child undernutrition in SSA countries [47, 48, 66].

The findings in our analysis have some policy and practice implications. First, the widening equity gap in child undernutrition over the 15 years implies that there is a critical need for intentional monitoring and targeting of disadvantaged segments of the population in the design and implementation of nutrition programs. Secondly, the current level of child undernutrition especially among the poor demands an urgent intervention to promote the well-being of affected individuals and families. Finally, special advocacy and behavior change communication interventions targeting female child feeding practice and women’s education, and customized policy action for urban child nutrition are possible actions to improve the inequality observed.

### Study limitation

The findings reported in this analysis may not reflect the current standing of population groups because it is more than five years since the last survey included in our study. However, the trends we have picked imply that changes in the pattern and magnitude of inequity in child undernutrition are unlikely unless there is a significant shift in how nutrition programs are designed and implemented. In addition, this study tried to examine the determinants of inequality using accessible variables on the DHS dataset, which limited us from exploring many variables of interest, including the socioeconomic and political context of the country. Future researchers in this same area should consider uncovering those variables.

## Conclusion

Despite encouraging reductions in child undernutrition rates for stunting and underweight, the prevalence of wasting has remained persistently high in Ethiopia since 2005. Moreover, wealth-related inequality in child undernutrition has increased for most of the undernutrition indicators. Social determinants of child undernutrition identified in this study warrant urgent implementation of strategies to reduce their health impacts in SSA.

## Abbreviations

DHS: Demographic and Health Survey
RR: Rate Ratio
SDG: : Sustainable Development Goals
SSA: sub-Sahara Africa
WHO: World Health Organization
